# Deep residual 2D convolutional neural network for cardiovascular disease classification

**DOI:** 10.1038/s41598-024-72382-3

**Published:** 2024-09-26

**Authors:** Haneen A. Elyamani, Mohammed A. Salem, Farid Melgani, N. M. Yhiea

**Affiliations:** 1 Department of Mathematics, Faculty of Science, Suez Canal University, Ismailia, 44745 Egypt; 2 Media Engineering and Technology, German University in Cairo (GUC), Cairo, Egypt; 3 Department of Information Engineering and Computer Science, University of Trento, Via Sommarive, 14, I-3812 Trento, Italy; 4 Faculty of Informatics and Computer Science, The British University in Egypt (BUE), Cairo, Egypt

**Keywords:** Computer science, Cardiovascular diseases, Cardiovascular diseases

## Abstract

Cardiovascular disease (CVD) continues to be a major global health concern, underscoring the need for advancements in medical care. The use of electrocardiograms (ECGs) is crucial for diagnosing cardiac conditions. However, the reliance on professional expertise for manual ECG interpretation poses challenges for expanding accessible healthcare, particularly in community hospitals. To address this, there is a growing interest in leveraging automated and AI-driven ECG analysis systems, which can enhance diagnostic accuracy and efficiency, making quality cardiac care more accessible to a broader population. In this study, we implemented a novel deep two-dimensional convolutional neural network (2D-CNN) on a dataset of PTB-XL for cardiac disorder detection. The studies were performed on 2, 5, and 23 classes of cardiovascular diseases. The our network in classifying healthy/sick patients achived an AUC of 95% and an average accuracy of 87.85%. In 5-classes classification, our model achieved an AUC of 93.46% with an average accuracy of 89.87%. In a more complex scenario involving classification into 23 different classes, the model achieved an AUC of 92.18% and an accuracy of 96.88%. According to the experimental results, our model obtained the best classification result compared to the other methods based on the same public dataset. This indicates that our method can aid healthcare professionals in the clinical analysis of ECGs, offering valuable assistance in diagnosing CVD and contributing to the advancement of computer-aided diagnosis technology.

## Introduction

Cardiovascular diseases (CVDs) refer to a class of disorders that involve the heart and blood vessels. CVDs are a major global health concern and a leading cause of mortality with approximately 17.9 million deaths recorded annually, representing 32% of all deaths globally^1^. Advances in technology, such as electrocardiography (ECG), ambulatory monitoring, and implantable devices, have greatly improved the ability to monitor and diagnose these conditions, contributing to more effective and targeted healthcare interventions.

ECG is a valuable tool for healthcare professionals to assess and monitor the electrical activity of the heart, aiding in the diagnosis and management of cardiovascular diseases. A standard ECG involves recording from 12 leads. The manual interpretation of ECG results by cardiologists faces significant challenges due to the diverse nature of heart diseases, each presenting unique ECG patterns. Recognizing these patterns requires extensive knowledge and experience, making it challenging for cardiologists to cover the entire spectrum effectively. Additionally, variations in heart signals among individuals, influenced by factors like age and race, contribute to the complexity. The similarity in ECG patterns across different heart conditions poses a risk of misdiagnosis or delayed diagnosis. Early detection of CVDs is a cornerstone of preventive cardiology. It enables healthcare providers to intervene before complications arise, tailor treatment plans, and improve the overall prognosis for individuals with these conditions. Given the rapid advancements in ECG technology and the limited number of cardiologists available, there is a growing interest in developing accurate and automated methods for diagnosing ECG signals. This has become a significant area of research for scientists, aiming to enhance the accuracy and effectiveness of cardiovascular diagnoses.

Traditional methods involve extracting handcrafted features like QRS complex, ST segment, and T wave characteristics^2^. Once these features are extracted, they are used as input to a machine learning model to classify heartbeats into different classes. Common machine learning algorithms like Support Vector Machines (SVMs)^3,4^, Random Forests^5^, k-Nearest Neighbors (k-NN)^6,7^, artificial neural network (ANN)^8,9^, or others may be employed for classification tasks. Deep neural networks (DNNs) have played a major role in achieving the state-of-the-art performance in various machine learning tasks, making them a central focus of research and development in the field of artificial intelligence. Automatic ECG analysis using DNNs has shown promising results in various clinical applications, enabling more accurate and efficient classification, detection, and diagnosis of cardiac conditions. Deep learning (DL) models, especially convolutional neural network (CNN) and recurrent neural network (RNN), have been utilized to extract features from ECG signals for tasks such as arrhythmia detection, heart disease diagnosis, and abnormality detection. CNNs are effective in capturing local patterns and spatial dependencies in the ECG signals, making them suitable for feature extraction. Some research has used one-dimensional convolutions^10,11^ and two-dimensional convolutions^12^ for ECG classification. RNN specifically created for handling sequential data, such ECG signals^13^ which includes Long-Short Term Memory (LSTM) network^14^ and bidirectional LSTM network^15^. Some models use hybrid architectures for example, combining CNN and RNN to capture spatio-temporal information^16,17^. Recently, the transformer has gained in popularity as a deep learning model, alongside CNN and RNN. In recent years, newer architectures such as transformers, which use a self-attention mechanism, have gained popularity for tasks involving sequential data, that allows the model to focus on different aspects of the ECG signal simultaneously^18,19^.

Applying DL methods to analyze ECG signals poses a difficulty for researchers, primarily due to the constrained availability of suitable datasets. Moreover, training DL models, especially large-scale architectures, requires substantial computational resources. Access to high-performance computing platforms may be a limiting factor for some researchers or healthcare institutions. The PTB-XL database emerged as a solution to address the scarcity of available data. This extensive online electrocardiography dataset was publicly released in April 2020. It serves as a valuable resource for researchers in the field. In this paper^21^ applied various algorithms from the literature based on CNN and RNN. The authors of^22^ suggested approach involves a deep learning architecture composed of a 33-layer CNN fed to a non-local convolutional block attention module (NCBAM). In another study, the authors^23^ structured model into two distinct components. In the initial phase, each channel of the input ECG recording is individually processed to produce a channel-specific encoding. The second phase, the model aggregates the separate encodings from each channel to make predictions or classifications. The authors of^24^ use DNN based on 2D-CNN for cardiovascular classification. In another study, the authors focused on studying one type of cardiovascular disease, which is atrial fibrillation (AF)^25^. They used diverse deep learning models to detect AF using ECG signals. Researchers continue to explore and refine deep learning techniques for ECG classification, aiming to enhance the reliability and generalizability of these models in clinical settings. This ongoing research is expected to have a substantial impact on the field of cardiology and improve patient care through more accurate and timely diagnoses.

Motivated by these challenges, we developed a novel and effective automated model (2D-CNN) for the classification of cardiovascular diseases. CNN is employed to capture features from the electrocardiogram signal. Each layer of the CNN is responsible for identifying distinct characteristics within the signal. By testing on the PTB-XL dataset^20^, our model demonstrated an accuracy of 87.85% in the classification of 2-classes, 89.87% in classification with 2-classes, and accuracy of accuracy of 96.88% in classification with 23-classes. In classification tasks, the highest achieved AUC score was 95% when distinguishing between 2-classes, while it decreased to less than 93.46% for 5-classes and 92.14% for 23-classes. Compared with existing state-of-the-art methods, our study improved the performance of ECG classification.

This paper is organized as follows. Section 2 outlines the dataset details and the architecture of the proposed 2D-CNN model. Section 3 discusses the experimental setting and evaluation metrics. The experimental results, analysis, and comparisons with other studies in the literature are presented in Section 4. Finally, Section 5 concludes the main point of the paper.

## Materials and methods

### PTB-XL dataset

**Figure 1:**
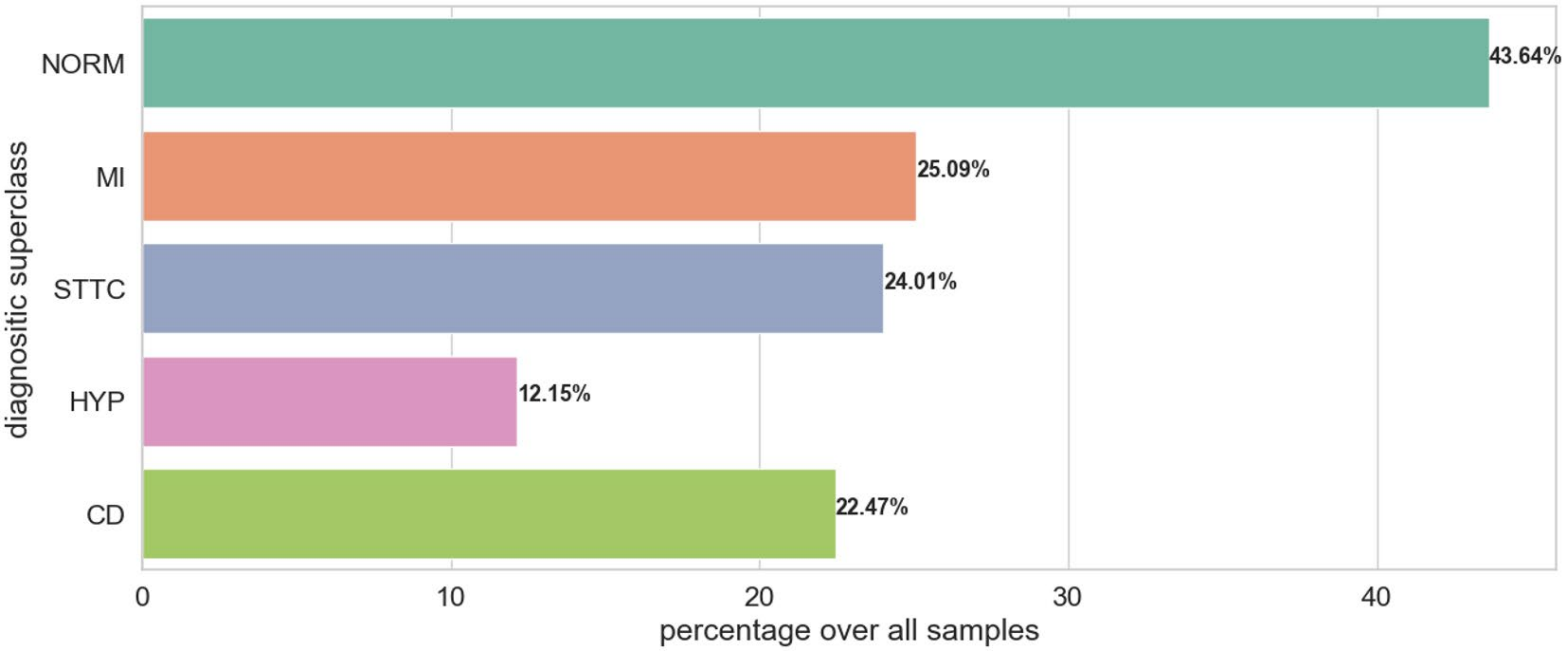
Distribution of superclasses in the PTB-XL dataset.

**Figure 2:**
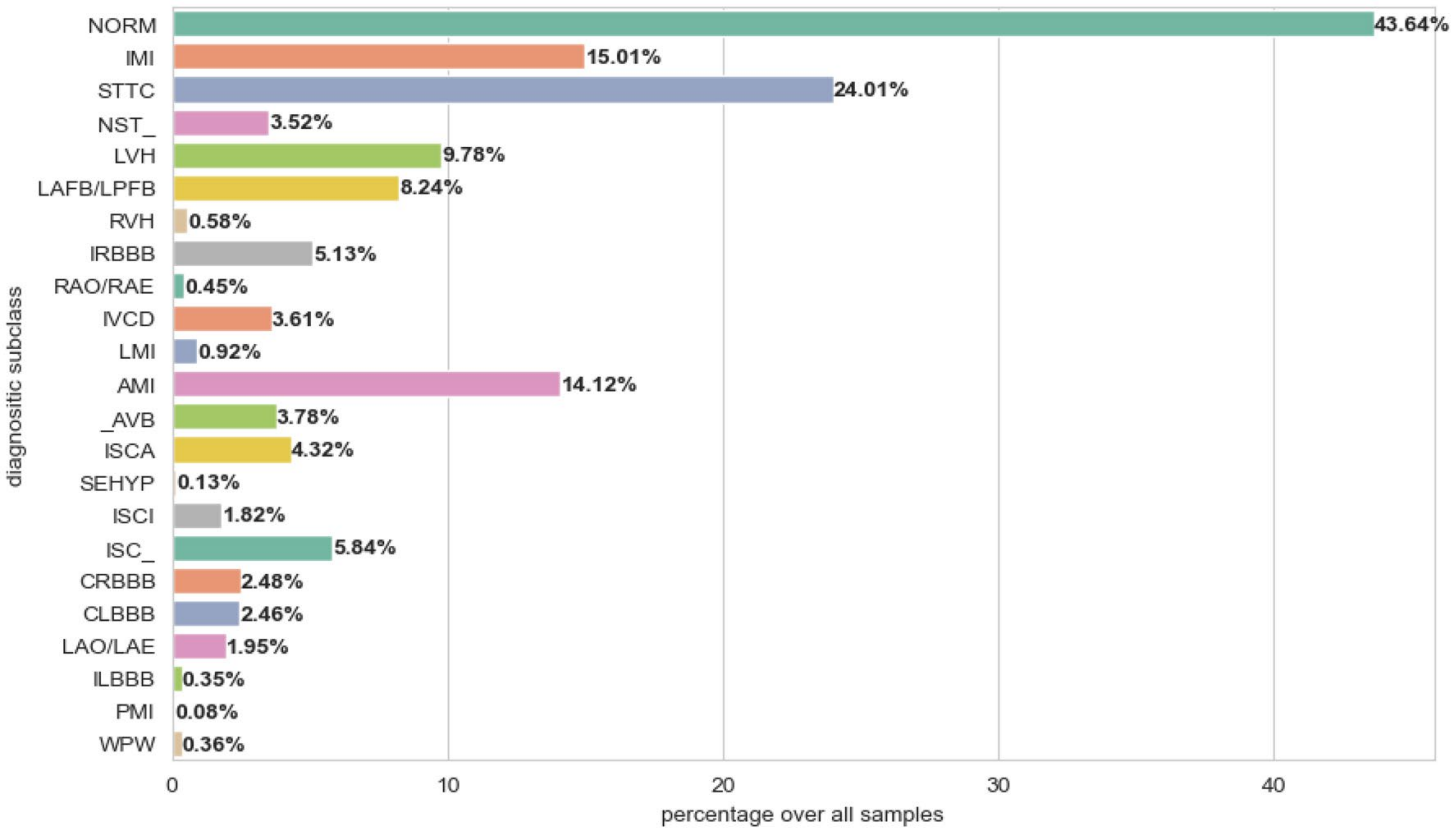
Distribution of subclasses in the PTB-XL dataset.

**Figure 3:**
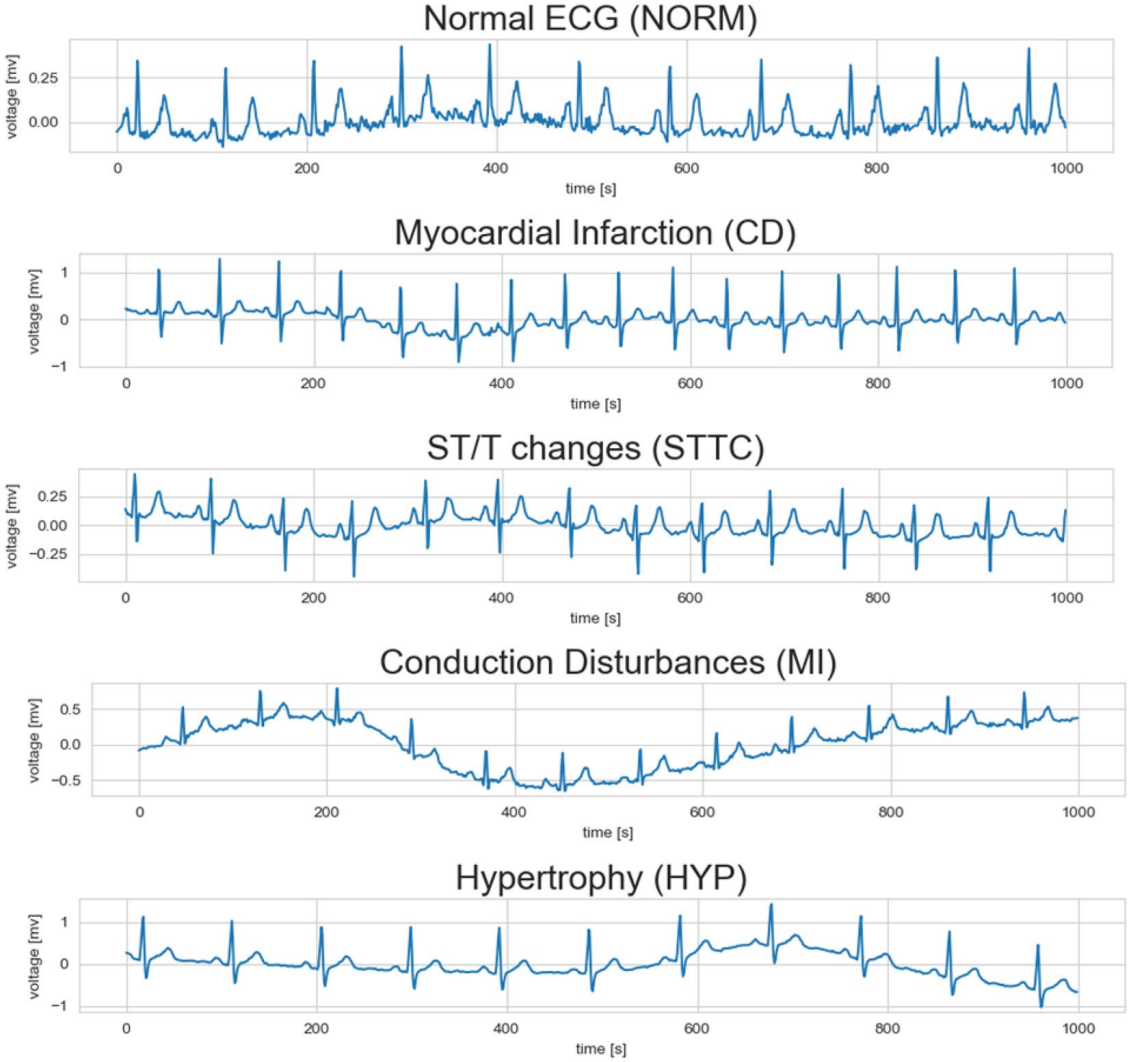
Examples of rhythm ECG signals using lead II.

The PTB-XL dataset is a publicly available dataset for research purposes in the field of electrocardiography (ECG)^20^. This dataset comprises 21799 12-lead recording collected from 18869 patients. The gender distribution is nearly balanced, with 48% female and 52% male patients. The ages of the patients span from 0 to 95 years. Every ECG recording was labeled with a diagnostic statement chosen from a total of 71 different diagnostic statements available in the dataset. These diagnostic statements were then grouped into five main pathologically relevant classes based on similar pathology. Table 1 presents a comprehensive overview of the primary 5-classes and their subclasses within the dataset. Figure 1 presents the distribution of diagnoses across the superclasses investigated. Meanwhile, Figure 2 displays the distribution of diagnostic subclasses, providing a more detailed breakdown of specific cardiac diagnoses within each superclass. Figure 3 shows samples of cardiac rhythms, consistent with the data contained in Table 1. The PTB-XL dataset includes ECG waveforms that have been sampled at both 500 Hz and 100 Hz. However, for all experiments, the ECG data sampled at 100 Hz is utilized.

**Table 1:** Overview of the superclassess and their subclasses in the PTB-XL dataset.

Number of Records	Description	Acronyms	Subclass (n)
9514	Normal ECG	NORM	Normal ECG (9514)
4898	Conduction Disturbances	CD	LAFB/LPFB (1797), IRBBB (1118) _AVB ( 823) , IVCD (787) CRBBB (541), CLBBB (536) WPW (79), LBBB (77)
5235	ST/T changes	STTC	STTC (2239), ISC_ (1272) ISCA (941) ,NST_ (767) ISCI (397)
5469	Myocardial Infarction	MI	AMI (3078) IMI (3271) LMI (201), PMI (17)
2649	Hypertrophy	HYP	LVH (2132), RAO/RAE (99) RVH (126), SEHYP (29) LAO/LAE (426)

### Proposed network architecture

**Figure 4:**
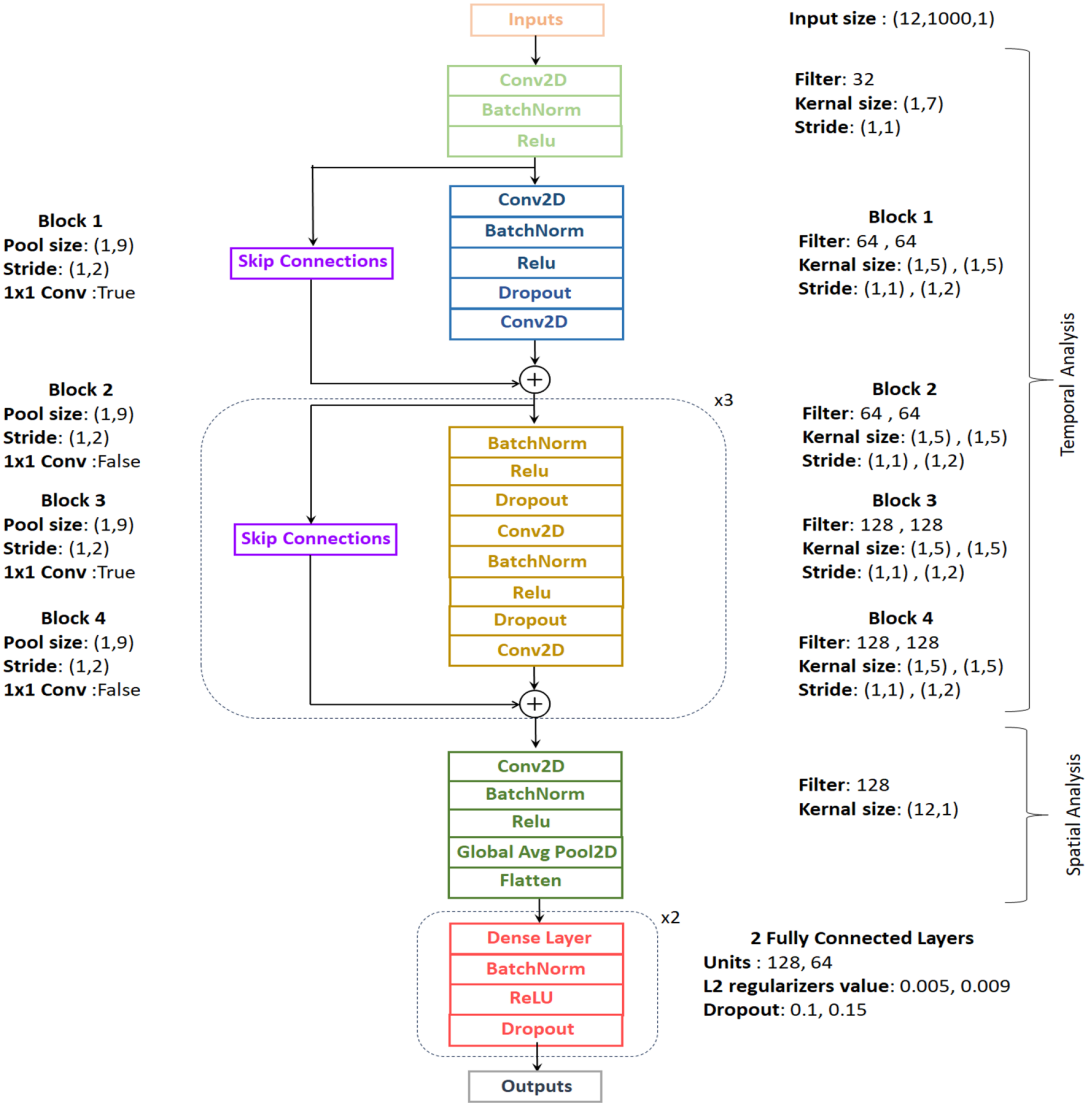
The proposed deep learning network architecture for automatic classification of cardiovascular diseases.

**Figure 5:**
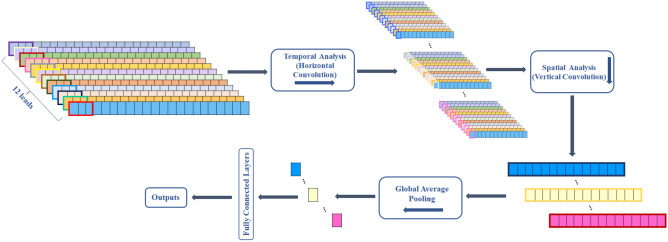
The process of extracting features in both temporal and spatial analyses from a signal.

We developed a convolutional neural network to detect cardiovascular diseases. Its architecture is shown in Figure 4. The network takes a time-series of raw ECG signals as input and produces a sequence of label predictions as output. This design enables the efficient training of CNNs through skip connections following a strategy similar to the residual network architecture^26^. The skip connections between neural network layers enhance training dynamics and performance, particularly in very deep networks, by allowing information to propagate effectively. The network architecture was adjusted to incorporate spatial and temporal feature extraction layers. Figure 5 illustrates the process of feature extraction in both temporal and spatial analysis on a signal. The network comprises a convolutional layer (Conv) followed by four stacked residual blocks, with each block containing two convolutional layers. Following the extraction of temporal features by the initial group of blocks, another spatial block was used to combine data from all leads, using a Conv layer followed by a global average pooling layer. A global average pooling layer is added between the final convolutional layer and the first fully connected (FC) layer to prevent overfitting. This addition improves model performance and reduces the number of model parameters. Afterwards, the extracted features of pooling were flattened and used in a fully connected (Dense) layer. The last layer of the network is a fully connected layer and was activated with a sigmoid function ($\sigma$). It contains a number of neurons corresponding to the possible classes the input could belong to. This choice is made because the classes are not mutually exclusive, meaning that two or more classes can be present in the same record. The sigmoid activation function is suitable for multi-label classification tasks, where each class can be independently activated.

The filter size of the Conv layers starts at 32 in the initial layer, increases to 64 in the first and second blocks, and then reaches 128 in the third and fourth blocks. This progression is designed to capture as much information as possible across the different CNN filters. The model uses a kernel size of 1 × 7 in the first convolutional layer, a kernel size of 1 × 5 for the first four residual blocks, and a kernel size of 12 × 1 in the last layer. The output of each Conv layer in the blocks is rescaled using batch normalization (BatchNorm)^27^ and fed into a rectified linear unit (ReLU) non-linearity^28^ and dropout^29^ with a probability of 0.1 to reduce overfitting and accelerate the training process. In skip connections, max pooling^30^ is used to reduce the size of the feature map, effectively summarizing key features and reduce computational complexity. To ensure dimensional alignment with the signals in the main branch, max pooling and 1$\times$1 Conv layers (also known as 1$\times$1 conv) are integrated into the skip connections in odd blocks. In even blocks, max pooling alone is sufficient.

## Experimental setup

### Used tools

The computations were performed on a Core i7 CPU-based system with 16GB of internal RAM, a 250GB external SSD hard drive along with an internal hard drive, and an NVIDIA 1050 GPU with 4GB of memory. In this research, TensorFlow, scikit-learn, NumPy, and Jupyter notebook environment were used to implement the neural networks. The model has 396,677 trainable parameters and 1,728 non-trainable parameters, with an average training time of 1.4 hours.

### Preprocessing

The PTB-XL dataset is provided in 10 folds by the dataset authors. This indicates that the dataset has been pre-divided into 10 subsets, each containing a specific portion of the data. In our experiments with the PTB-XL dataset, we utilized data from the initial nine folds for both training (88%) and validation (12%). Subsequently, we reserved the data from the tenth fold exclusively for testing purposes. We aimed to classify diagnoses into 2, 5, and 23 classes. For the classifications including the 5 and 23 classes, some records had multiple labels. These labels were One-Hot encoded, with each diagnosis represented as a bit in a 5-bit and 23-bit array, respectively. In our study, we chose not to use data augmentation techniques and instead relied solely on the inherent power of the 2D CNN model we proposed.

### Ablation study

In our classification setup, the 2-class classification task distinguishes between “normal” and “abnormal” heartbeats. The “normal” class includes instances labeled as “NORM” while the “abnormal” class comprises instances from the “MI”, “STTC”, “CD”, “HYP”, and “OTHER” subclasses. The “OTHER” class encompasses signals that do not belong to the five main subclasses. In the 5-class classification task, the classes are defined as “MI”, “STTC”, “CD”, “HYP”, and “NORM” with each representing a specific type of abnormality or the normal state. Figure 2 illustrates the 23-classes used in the classification task.

We experimented our model by with various combinations of leads to determine the best model related to heart disease. Selected channel combinations include lead I, bipolar limb leads include (I, II, and III), unipolar limb leads consist of (AVR, AVL, and AVF), limb leads are formed by combining bipolar and unipolar limb leads, and precordial leads comprise (V1-V6). Furthermore, all twelve available leads in the ECG recording are considered (I, II, III, AVR, AVL, AVF, V1-V6).

### Parameter setting

The network was trained from scratch, starting with the random initialization of weights. We used the Adam optimization algorithm^31^ to update the weights with momentum value of 0.9 and mini batch of size 32. Initially, the learning rate was set to 0.0005. This value has been reduced by a factor of 10 whenever there is no improvement in the validation loss for three consecutive epochs. Training is done over 30 epochs, and the final model is selected based on the optimal validation results in which the lowest error was achieved during the optimization process.

In general, we selected the hyper-parameters and optimization algorithm for our architecture using a combination of grid search and manual tuning. For the architecture, we focused on exploring the number of Conv layers, the size and number of Conv filters, and the use of skip connections. We found that skip connections were useful when the block had two Conv layers. Additionally, we adjusted the learning rate if no performance improvement was observed over three consecutive epochs to ensure the fastest convergence.

### Evaluation metrics

To evaluate our method, we use the standard metrics for heartbeat classification techniques^12^. Calculations for these metrics are presented in the Eqs (1-5) described below:

1 $$\begin{aligned} & Accuracy= \dfrac{TP + TN}{TP + FP + TN + FN} \end{aligned}$$

2 $$\begin{aligned} & Precision= \dfrac{TP}{TP + FP} \end{aligned}$$

3 $$\begin{aligned} & Recall (sensitivity)= \dfrac{TP}{TP + FN} \end{aligned}$$

4 $$\begin{aligned} & F1score= 2 \times \dfrac{Precision\times Recall}{Precision + Recall} \end{aligned}$$

The AUC (Area Under the Curve) measures the class separability of a classification model by plotting the Receiver Operating Characteristic (ROC) curve, which shows the True Positive Rate (TPR) on the y-axis and the False Positive Rate (FPR) on the x-axis at various classification thresholds.

5 $$\begin{aligned} TPR (Recall)= \dfrac{TP}{TP + FN} , \quad FPR= \dfrac{FP}{TN + FP} \end{aligned}$$

The AUC-ROC curve ranges from 0 to 1. Generally, a higher AUC indicates better model performance, with values closer to 1 representing excellent class separability and values closer to 0.5 suggesting poor separability. Where, TP (True Positive) represents instances where the model correctly identifies cases of a specific cardiac condition. TN (True Negative) represents instances where the model correctly identifies a normal ECG. FP (False Positive) represents instances where the model incorrectly predicts the presence of a specific cardiac condition when it is not actually present. FN (False Negative) represents instances where the model fails to detect a cardiac condition when it is present. While accuracy is a simple and intuitive metric, it can be deceptive when confronted with imbalanced classes^32^. By considering metrics like Precision, Recall, F1-score, and AUC alongside accuracy, one can gain a more accurate understanding of the classification model performance. These metrics provide important insights into the ability of classifier performance to differentiate between classes, making them essential tools for evaluating classifier performance in real-world applications.

## Results and analysis

**Figure 6:**
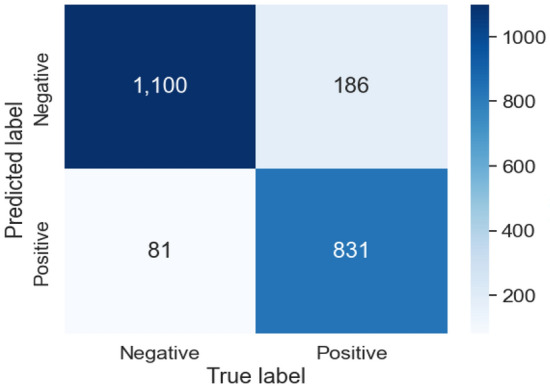
Confusion matrices for our method on test dataset for 2 classes.

**Figure 7:**
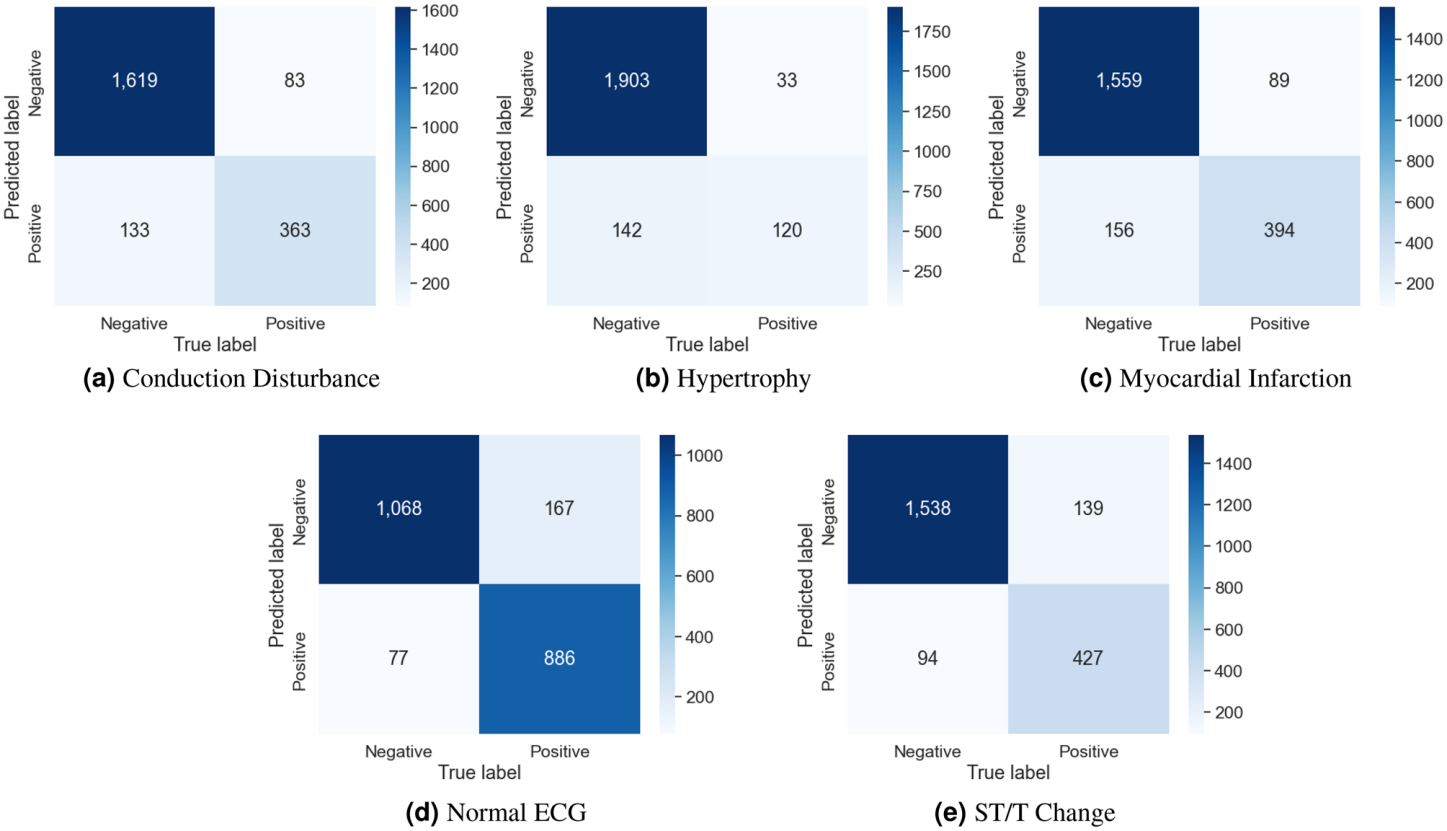
Confusion matrices for our method on test dataset for 5 classes.

**Figure 8:**
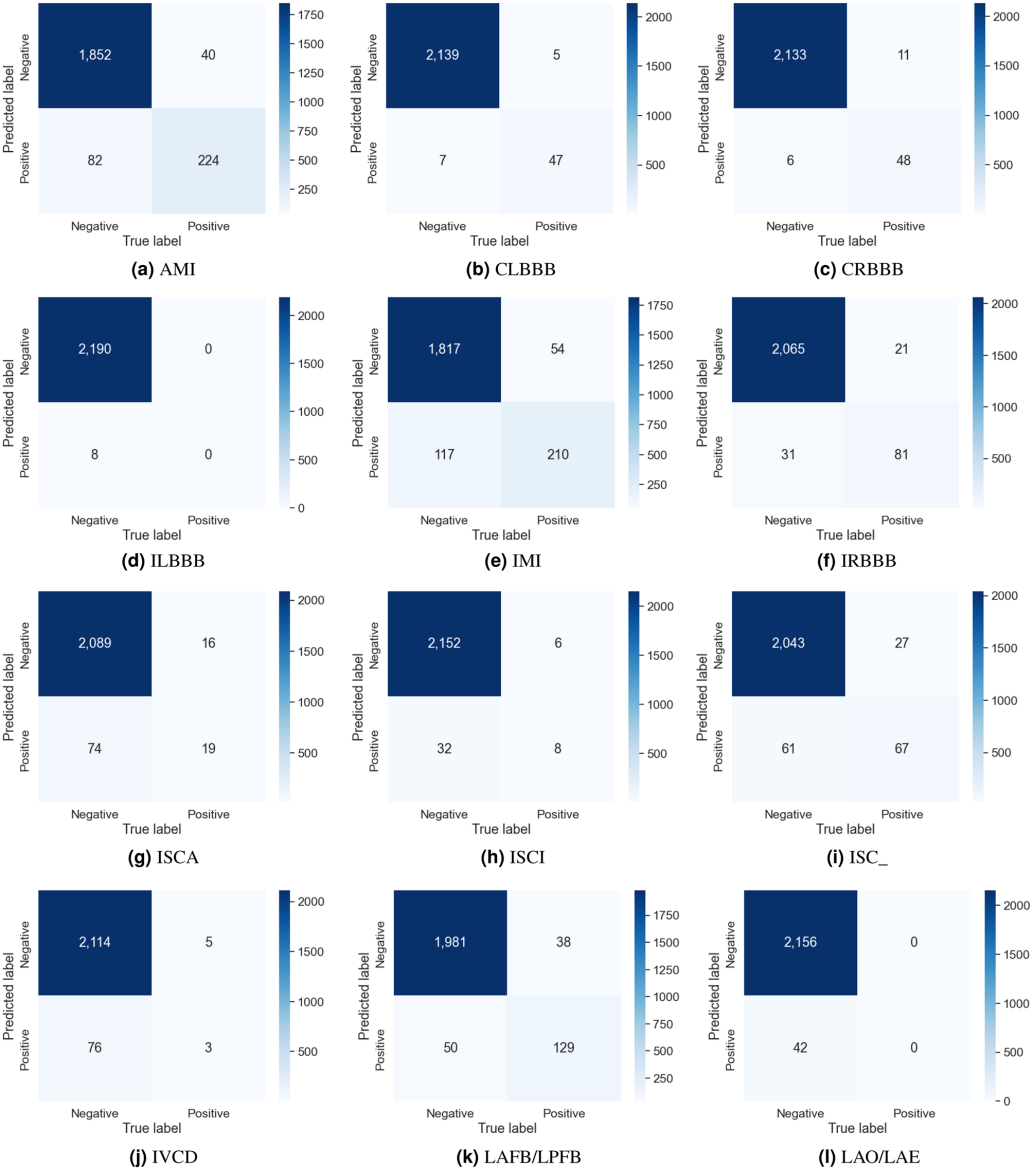
The first part of the confusion matrices depicting the performance of our method on the test dataset for 23 classes.

**Figure 9:**
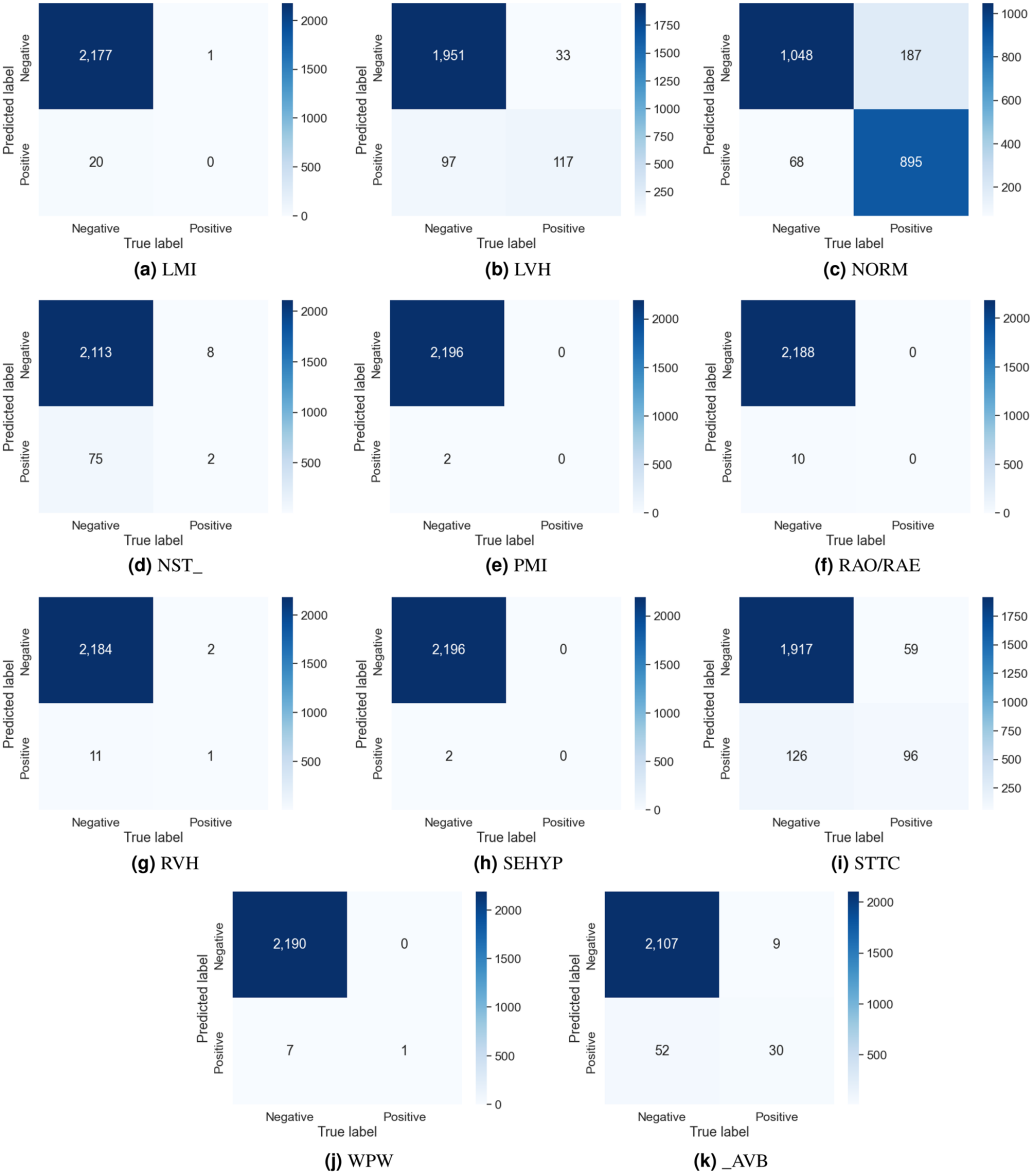
The second part of the confusion matrices depicting the performance of our method on the test dataset for 23 classes.

The evaluation results for the different channel combinations are presented in Tables 2, 3, and 4 for tasks involving the classification of 2, 5, and 23 different heart disease classes, respectively. The data indicates that the performance metrics are at their peak when all 12-channels in the ECG recording are utilized for overall, surpassing the results obtained from various channel combinations. In both the 2-class and 5-class classification tasks, the precordial leads demonstrated the second-highest overall performance. But in the 23-class classification task, the precordial leads demonstrated superior performance across all metrics except for AUC, where the limb leads showed slightly better results. Among the groups, the unipolar limb leads were considered the least effective. In 5-class classification, Tables 5 and 6 provide details about the AUC and the accuracy for each class scores for various combinations of channels. Notably, disorder classes such as CD and HYP showed superior classification metrics. Furthermore, Tables 7 and 8 also provide a detailed analysis of AUC and accuracy scores for individual classes across various channel combinations in the 23-class classification. The model achieves its best performance in the subclass CLBBB, while the lowest performance is observed in the LAO/LAE subclass. Confusion matrices analyzing the performance of our method on a test dataset are shown in Figures 6, 7, 8, and 9. Classification accuracy for two classes typically remains consistent across different classifier types. However, for subclasses with fewer records, skipping can happen, impacting the model skewness. This explains the decreased classification performance observed with larger class sizes, such as with 5 and 23 classes. Classifiers may have difficulty learning the distinguishing features of subclasses that have fewer records due to limited data. This commonly leads to reduced performance for these underrepresented classes. In Figure 7, When a class like NORM is the most numerous, the model is exposed to more examples of this class during training. Consequently, the model learns to recognize this class more effectively, leading to higher accuracy for that class compared to others. In a different situation, Figures 8, and 9 show that a significant portion of the misclassification is due to an imbalanced dataset. Classes with fewer records (such as ILBBB, LAO/LAE, LMI, PMI, RAO/RAE, and SEHYP) are less commonly selected by the model, which resulted in no accurate positive predictions (true positives) within these subclasses. In brief, the standard 12-lead ECG setup offers the most superior performance. With the removal of leads, there is a consequent decline in performance due to the vital information lost in the channels. This highlights the pivotal role of utilizing multi-channel data in the diagnosis of heart conditions. In addition, class imbalance can significantly affect classification accuracy, especially when dealing with subclasses that have a small number of records.

**Figure 10:**
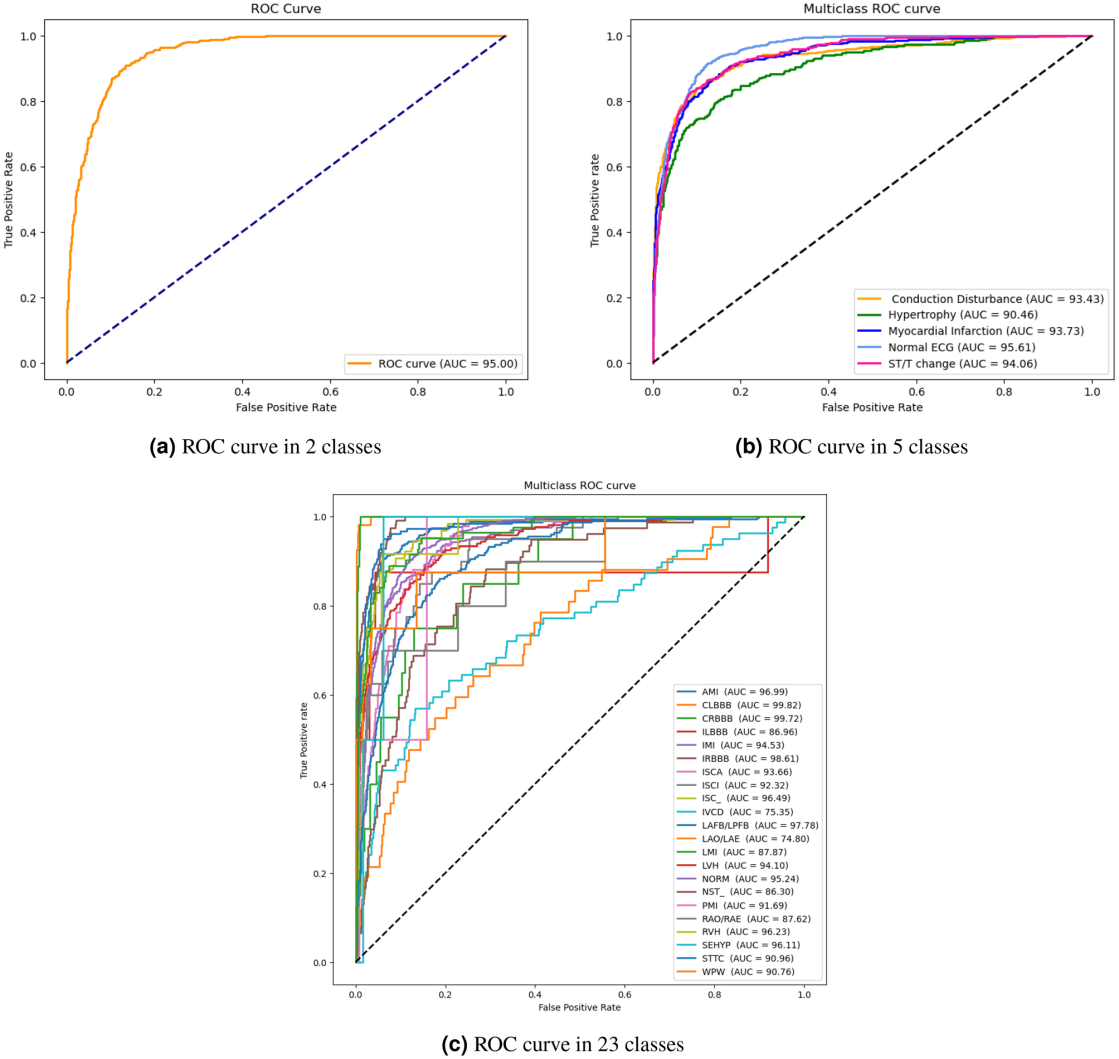
ROC curves for 2, 5, and 23 classes.

Results are grouped by other studies in the literature and number of classes. All other studies were trained on the PTB-XL dataset for classify 2 and 23 classes due to the scarcity of previous research on the same problem. Table 9 displays the results of the proposed network and compares them with the other studies in two classes classification. Our method achieves an accuracy and AUC score of 87.85%, 95%, respectively, for the detection normal and abnormal heartbeat. The proposed method obtains higher classification results than the other studies in classifying 2-classes. The highest AUC value produced by other models reaches 94.47%, which is lower than 95%. The comparison of our network with other relevant methods in literature in classifying 5-classes is given in Table 10. Our network demonstrates better performance in cardiovascular disease classification compared to previously published experimental results. In addition, our proposed network scored an accuracy rate of 89.87%, an AUC of 93.46%, and a micro F1 score of 79.74% for the detection of heartbeat on test dataset. This represents enhancements of 0.14%, 0.05%, and 0.46%, respectively, compared to the best earlier state-of-the-art results^24^, which reached an accuracy of 89.73%, AUC of 93.41%, and micro F1 score of 79.28%. Table 11 show the results obtained from our model and compares them with previous studies on 23-class classification tasks. Our model achieves accuracy and AUC scores of 96.88% and 92.18%, respectively. The highest AUC value achieved by competing models is 91.93%, which falls short of our model AUC score of 92.18%. The ROC curves for 2, 5, and 23 classes are shown in the Figure 10. Our proposed method consistently demonstrates superior performance across various classification tasks compared to the state-of-the-art methodologies in cardiovascular disease classification

**Table 2:** Overall performance of our model for various ECG leads for 2 classes.

Channels	Accuracy	Precision	Recall	Micro F1	AUC
**Leads I**	78.66	78.66	78.66	78.66	87.07
**Bipolar limb leads**	84.39	84.39	84.39	84.39	91.88
**Unipolar limb leads**	58.51	58.50	58.50	58.51	52.09
**Limb leads**	84.08	84.07	84.07	84.08	91.62
**Precordial leads**	85.17	85.16	85.16	85.17	93.10
**12 leads**	**87.85**	**87.85**	**87.85**	**87.85**	**95.00**

**Table 3:** Overall performance of our model for various ECG leads for 5 classes.

Channels	Accuracy	Precision	Recall	Micro F1	AUC
**Leads I**	83.00	69.84	58.23	63.52	83.19
**Bipolar limb leads**	86.40	75.26	69.19	72.10	89.06
**Unipolar limb leads**	63.88	29.66	30.76	30.20	58.18
**Limb leads**	86.81	76.33	69.66	72.85	89.26
**Precordial leads**	88.16	79.04	72.67	75.72	91.00
**12 leads**	**89.87**	**81.08**	**78.43**	**79.74**	**93.46**

**Table 4:** Overall performance of our model for various ECG leads for 23 classes.

Channels	Accuracy	Precision	Recall	Micro F1	AUC
**Leads I**	95.35	70.19	39.51	51.09	85.66
**Bipolar limb leads**	96.10	74.35	53.52	62.25	90.40
**Unipolar limb leads**	91.21	22.81	19.47	21.02	66.18
**Limb leads**	96.13	74.12	54.58	62.87	89.60
**Precordial leads**	96.36	75.67	57.94	65.63	89.52
**12 leads**	**96.88**	**79.12**	**65.19**	**71.49**	**92.18**

**Table 5:** Macro ROC-AUC scores comparison on various combinations ECG leads for 5 classes.

Channels	CD	HYP	MI	NORM	STTC	AUC
**Leads I**	81.19	80.43	78.13	88.70	87.08	83.19
**Bipolar limb leads**	87.86	85.93	88.15	92.89	91.03	89.06
**Unipolar limb leads**	71.78	75.76	60.41	61.51	53.24	58.18
**Limb leads**	87.50	84.89	87.43	92.59	90.60	89.26
**Precordial leads**	89.96	88.67	8751	93.47	92.91	91.00
**12 leads**	**93.43**	**90.46**	**93.73**	**95.60**	**94.05**	**93.46**

**Table 6:** Accuracy scores comparison on various combinations ECG leads for 5 classes.

Channels	CD	HYP	MI	NORM	STTC	Accuracy
**Leads I**	84.44	89.30	78.38	80.39	83.34	83.00
**Bipolar limb leads**	87.39	89.94	84.30	84.16	85.21	86.40
**Unipolar limb leads**	77.79	88.44	26.88	56.27	76.02	63.88
**Limb leads**	87.07	89.89	83.71	84.07	85.25	86.81
**Precordial leads**	87.67	90.80	85.16	86.53	88.67	88.16
**12 leads**	**90.17**	**92.03**	**88.85**	**88.89**	**89.39**	**89.87**

**Table 7:** Macro ROC-AUC scores comparison on various combinations ECG leads for 23 classes.

Channels	Leads I	Bipolar limb leads	Unipolar limb leads	Limb leads	Precordial leads	12 leads
**AMI**	85	86.44	71.31	86.62	96.89	96.98
**CLBBB**	99.69	99.63	83.50	99.62	99.77	99.81
**CRBBB**	99	99.47	59.68	99.64	99.71	99.71
**ILBBB**	89.94	92.23	52.51	88.22	94.77	86.95
**IMI**	72.82	93.70	73.57	94.33	80.83	94.53
**IRBBB**	77.75	84.49	65.14	83.51	98.41	98.60
**ISCA**	88.89	89.92	39.85	90.13	92.96	93.65
**ISCI**	73.19	91.23	62.39	94.38	84.26	92.32
**ISC**_	91.42	93.90	49.91	93.57	96.09	96.49
**IVCD**	72.02	71.68	54.58	73.07	74.02	75.35
**LAFB/LPFB**	79.73	97.83	79.88	97.97	90.99	97.77
**LAO/LAE**	72.98	76.31	60.13	74.84	75.20	74.79
**LMI**	88.27	85.42	74.41	82.29	78.74	87.86
**LVH**	85.42	88.96	60.52	87.50	91.82	94.10
**NORM**	89	92.99	56.33	93.02	93.68	95.23
**NST**_	84.67	85.36	50.96	85.54	82.97	86.29
**PMI**	86.29	93.26	73.49	82.46	86.06	91.68
**RAO/RAE**	76.75	93.59	83.34	84.39	84.98	87.61
**RVH**	92.44	95.66	61.87	95.85	92.21	96.22
**SEHYP**	96.74	98.33	91.73	99.04	86.49	96.10
**STTC**	82.17	87.24	60.38	86.45	90.84	90.95
**WPW**	91.30	85	76.75	91.42	90.79	90.76
**_AVB**	94.60	96.53	79.85	96.87	96.38	96.25
**ROC-AUC**	**85.66**	**90.40**	**66.18**	**89.60**	**89.52**	**92.18**

**Table 8:** Accuracy scores comparison on various combinations ECG leads for 5 classes.

Channels	Leads I	Bipolar limb leads	Unipolar limb leads	Limb leads	Precordial leads	12 leads
**AMI**	87.35	88.08	66.24	88.58	94.58	94.44
**CLBBB**	99.27	99.18	97.54	99.22	99.49	99.45
**CRBBB**	98.99	98.95	93.90	98.90	99.13	99.22
**ILBBB**	99.63	99.63	99.63	99.63	99.63	99.63
**IMI**	85.16	90.76	51.41	91.71	85.89	92.22
**IRBBB**	94.94	95.08	94.90	94.85	97.04	97.63
**ISCA**	95.85	95.67	95.76	95.67	95.58	95.90
**ISCI**	98.18	98.40	97.67	98.27	98.18	98.27
**ISC**_	94.49	95.31	94.13	95.22	95.95	95.99
**IVCD**	96.40	96.40	96.31	96.31	96.36	96.31
**LAFB/LPFB**	92.44	95.99	86.12	96.13	93.90	95.99
**LAO/LAE**	98.08	98.08	98.08	98.08	98.08	98.08
**LMI**	99.09	99.09	99.09	98.95	99.09	99.04
**LVH**	91.03	91.67	90.26	91.85	93.63	94.08
**NORM**	80.66	85.75	56.18	85.80	86.16	88.39
**NST**_	96.45	96.54	96.49	96.31	96.31	96.22
**PMI**	99.90	99.90	99.90	99.90	99.90	99.90
**RAO/RAE**	99.54	99.54	99.54	99.54	99.54	99.54
**RVH**	99.45	99.45	99.45	99.36	99.31	99.40
**SEHYP**	99.90	99.90	99.90	99.90	99.90	99.90
**STTC**	89.94	90.03	89.89	90.08	92.22	91.58
**WPW**	99.72	99.63	99.63	99.72	99.63	99.68
_**AVB**	9636	97.17	95.76	96.90	96.63	97.22
**Accuracy**	**95.35**	**96.10**	**91.21**	**96.13**	**96.36**	**96.88**

**Table 9:** Comparative results for the proposed method and other methods for 2 classes.

Methods	AUC	Accuracy
He *et al*. (ResNet-101)^33^	92.18	82.21
Mousavi *et al*.^34^	90.87	82.39
Hannun *et al*.^35^	93.58	85.03
Attia *et al*.^36^	93.29	84.03
Sharma *et al*.^37^	89.65	81.94
Anand *et al*.^24^	94.47	87.53
**Our method**	**95.00**	**87.85**

**Table 10:** Comparative results for the proposed method and other methods for 5 classes.

Methods	AUC	Accuracy
Karthik *et al*.^38^	-	88.24
Strodthoff *et al*.^21^	93.0	-
Zhang *et al*.^39^	93.0	-
Wang *et al*.^22^	93.14	-
Mehari *et al*.^40^	92.9	-
Li *et al*.^41^	93.1	-
Wen *et al*.^42^	92.97	-
Reddy *et al*.^23^	92.16	88.85
Anand *et al*.^24^	93.41	89.73
Cheng *et al*.^43^	92.33	89.80
Qiang *et al*.^44^	91.67	88.81
Huang *et al*.^45^	92.77	89.03
**Our method**	**93.46**	**89.87**

**Table 11:** Comparative results for the proposed method and other methods for 23 classes.

Methods	AUC	Accuracy
He *et al*. (ResNet-101)^33^	91.92	96.56
Mousavi *et al*.^34^	89.98	96.28
Hannun *et al*.^35^	90.86	96.41
Attia *et al*.^36^	89.02	96.64
Sharma *et al*.^37^	83.19	95.89
Anand *et al*.^24^	91.93	96.87
**Our method**	**92.18**	**96.88**

## Conclusions

In this article, we proposed an efficient method for heart disease classification using 2D convolutional neural networks. The research was performed on the recently published PTB-XL dataset to evaluate the performance of our model. The ablation study aims to understand how different combinations of ECG channels affect model performance. This study illustrated that using all 12-leads gives the best classification results. In our research, the results validated that the proposed model outperforms the existing state-of-the-art models by achieving the highest accuracy of 87.85%, 89.87% and 96.88% for 2, 5, and 23 classes, respectively. Furthermore, we achieved the highest AUC of 95% in recognizing 2-classes, while the AUC was below 93.46% for 5-classes and 92.18% for 23-classes. This indicates that the model discrimination capability tends to diminish as the number of classes increases, resulting in slightly lower AUC scores for more complex classification scenarios. Experimental results show that our model can effectively recognize different classes of cardiovascular diseases. This model can assist healthcare providers in making more informed decisions and potentially lead to earlier diagnosis and intervention in some cases. This study represents an initial investigation into the proposed 2D-CNN model. Future work will include exploring the impact of various data augmentation techniques. For this phase, we aimed to establish a strong foundational understanding of the model performance.

## Data Availability

The PTB-XL ECG dataset used and/or analyzed during the current study are available at https://physionet.org/content/ptb-xl/1.0.3/.^46^ (accessed on 11 August 2024).

## Code Availability

The code for training and evaluating the DNN model, and, also, the weighting of the best results presented in this paper, is available at: GitHub https://github.com/HaneenElyamani/ECG-classification.
